# Development and implementation of high-throughput SNP genotyping in barley

**DOI:** 10.1186/1471-2164-10-582

**Published:** 2009-12-04

**Authors:** Timothy J Close, Prasanna R Bhat, Stefano Lonardi, Yonghui Wu, Nils Rostoks, Luke Ramsay, Arnis Druka, Nils Stein, Jan T Svensson, Steve Wanamaker, Serdar Bozdag, Mikeal L Roose, Matthew J Moscou, Shiaoman Chao, Rajeev K Varshney, Péter Szűcs, Kazuhiro Sato, Patrick M Hayes, David E Matthews, Andris Kleinhofs, Gary J Muehlbauer, Joseph DeYoung, David F Marshall, Kavitha Madishetty, Raymond D Fenton, Pascal Condamine, Andreas Graner, Robbie Waugh

**Affiliations:** 1 Department of Botany & Plant Sciences, University of California (UCR), Riverside, CA, 92521, USA; 2 Department of Computer Sciences, University of California (UCR), Riverside, CA, 92521, USA; 3 Scottish Crop Research Institute (SCRI), Invergowrie, Dundee, DD2 5DA, UK; 4 Leibniz Institute of Plant Genetics and Crop Plant Research (IPK), Corrensstrasse 3, D-06466, Gatersleben, Germany; 5 USDA-ARS Biosciences Research Lab, Fargo, ND, 58105-5674, USA; 6 Department of Crop and Soil Science, Oregon State University, Corvallis, OR, 97331, USA; 7 Research Institute for Bioresources, Okayama University, Kurashiki, 710-0046, Japan; 8 USDA-ARS, Cornell University, Ithaca, NY, 14853, USA; 9 Department of Crop and Soil Sciences, Washington State University, Pullman, WA, 99164, USA; 10 Department of Agronomy and Plant Genetics, University of Minnesota, St. Paul, MN, 55108, USA; 11 Southern California Genotyping Consortium, University of California, Los Angeles, CA, 90095, USA; 12 Monsanto Research Centre, Bangalore, 560092, India; 13 Google, Mountain View, CA, 94043, USA; 14 Faculty of Biology, University of Latvia, 4 Kronvalda Boulevard, Riga, LV-1586, Latvia; 15 University of Copenhagen, Frederiksberg C, DK-1871, Denmark; 16 NIH National Cancer Institute, Neuro-Oncology Branch, Bethesda, MD, 20892, USA; 17 Department of Plant Pathology, Iowa State University, Ames, Iowa, 50011, USA; 18 International Crops Research Institute for the Semi-Arid Tropics (ICRISAT), Patancheru - 502 324, Andhra Pradesh, India; 19 NetSocial Marketing, Le Puech, 15600 Montmurat, France

## Abstract

**Background:**

High density genetic maps of plants have, nearly without exception, made use of marker datasets containing missing or questionable genotype calls derived from a variety of genic and non-genic or anonymous markers, and been presented as a single linear order of genetic loci for each linkage group. The consequences of missing or erroneous data include falsely separated markers, expansion of cM distances and incorrect marker order. These imperfections are amplified in consensus maps and problematic when fine resolution is critical including comparative genome analyses and map-based cloning. Here we provide a new paradigm, a high-density consensus genetic map of barley based only on complete and error-free datasets and genic markers, represented accurately by graphs and approximately by a best-fit linear order, and supported by a readily available SNP genotyping resource.

**Results:**

Approximately 22,000 SNPs were identified from barley ESTs and sequenced amplicons; 4,596 of them were tested for performance in three pilot phase Illumina GoldenGate assays. Data from three barley doubled haploid mapping populations supported the production of an initial consensus map. Over 200 germplasm selections, principally European and US breeding material, were used to estimate minor allele frequency (MAF) for each SNP. We selected 3,072 of these tested SNPs based on technical performance, map location, MAF and biological interest to fill two 1536-SNP "production" assays (BOPA1 and BOPA2), which were made available to the barley genetics community. Data were added using BOPA1 from a fourth mapping population to yield a consensus map containing 2,943 SNP loci in 975 marker bins covering a genetic distance of 1099 cM.

**Conclusion:**

The unprecedented density of genic markers and marker bins enabled a high resolution comparison of the genomes of barley and rice. Low recombination in pericentric regions is evident from bins containing many more than the average number of markers, meaning that a large number of genes are recombinationally locked into the genetic centromeric regions of several barley chromosomes. Examination of US breeding germplasm illustrated the usefulness of BOPA1 and BOPA2 in that they provide excellent marker density and sensitivity for detection of minor alleles in this genetically narrow material.

## Background

Complete genome sequences of many plants, including economically important small grain cereals such as barley, are unlikely to be available in the near future if they have large genomes and contain much repetitive DNA. The barley genome is 5200 Mbp, which is more than twelve times rice, and composed of at least 80% highly repetitive DNA, which is likely to preclude a whole-genome assembly from shotgun sequences obtained with currently available technologies. However, access to most of the genes of barley and numerous other organisms can be gained through cDNAs (generally expressed sequence tags; ESTs) and sequenced PCR amplicons, which provide a facile route to single nucleotide polymorphisms (SNPs) in protein-encoding transcribed genes. As of the January 2, 2009 release of dbEST, there were 525,527 Sanger-sequenced ESTs from barley. These were derived principally from eight malting barley cultivars and one wild barley accession, with a minor fraction from several other barley genotypes. Here we describe the use of the majority of this transcriptome sequence resource to develop high-throughput SNP genotyping in barley, application of the new SNP methods to the production of a high-density and high quality SNP map that can be related readily to prior maps through shared markers and other grass genomes through synteny, and deployment of these new resources in support of marker-assisted breeding and association genetic analyses.

In recent years there has been a surge in marker density and convergence toward consensus maps for barley. Rostoks et al. [1] developed a consensus map containing 1230 markers (RFLP, AFLP, SSR, SNP) from three doubled haploid populations. Wenzl et al. [2] combined DArT with RFLP, SSR and STS from nine mapping populations to create a consensus map containing 2935 markers. Marcel et al. [3] compiled RFLP, AFLP and SSR data from six mapping populations to produce a consensus map containing 3458 markers. Stein et al. [4] used three doubled haploid mapping populations and combined new data from 1,055 markers (RFLP, SSR, SNP) with prior data from 200 anchor markers to produce a 1255 marker consensus map. Varshney et al. [5] produced a 775 SSR consensus map by joining six independent maps. Potokina et al. [6] combined SNP and other transcript derived markers to position 1596 loci on the Steptoe × Morex [7] linkage map. Hearnden et al. [8] combined 1000 SSR and DArT markers on a map from a wide cross. Several additional maps which have used portions of the SNP data described in the present work have been published or are nearing publication including a 2890 SNP and STS map from the Haruno Nijo × OUH602 population [9] and a 2383 marker map (DArT, SNP, SSR, AFLP, RFLP, STS, QTL) from the Oregon Wolfe Barley population [10], among others. Marker intersection between these maps is significant, but missing data, non-uniform data quality and anonymity of many markers constrain the accuracy of the map merging process and the resolution of synteny between barley and other genomes. Here we describe a new element of the map convergence equation, a high fidelity and dense consensus map produced entirely from transcribed gene SNPs using only a very robust portion of genotyping data derived from four mapping populations utilizing the Illumina GoldenGate assay (Illumina Inc., San Diego, CA). Maps that include SNPs in protein-coding genes facilitate genome content comparisons by virtue of the high conservation of protein sequences across genera, thus enabling sequence similarity searches to find orthologs. The SNPs and data described herein have been made available incrementally in parallel with their production since mid-2005 to the barley community to facilitate research. Here we provide full details of the development of the SNP genotyping platform and some of the insight it has brought.

## Results and Discussion

### Identification of SNPs and development of GoldenGate Assays

**Figure 1:**
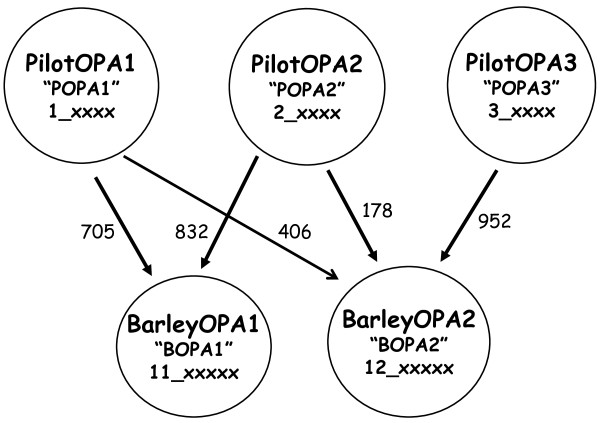
**Five 1536-plex GoldenGate assays**. The numbers of SNPs selected from each Pilot OPA (POPA1, POPA2, POPA3) for the design of each production scale OPA (BOPA1, BOPA2) are indicted next to the arrows connecting the pilot and production OPAs. See Supplemental Text (Additional File 1) for complete details.

Details of the identification of approximately 22,000 SNPs from EST and PCR amplicon sequence alignments, and development of three test phase and two production scale Illumina GoldenGate oligonucleotide pool assays (OPAs), are briefly summarized in Methods and provided more fully in Supplemental Text (Additional File 1). In total, 4596 SNPs were tested using 576 DNA samples on pilot OPAs POPA1 and POPA2, and 480 DNA samples on POPA3, followed by selection of 3072 technically satisfactory and genetically most informative SNPs for representation on two production OPAs (BOPA1, BOPA2) (Figure 1). Of these 4596 SNPs, 3456 originated from ESTs and 1140 from PCR amplicons derived from genomic sequences. Of the 3072 SNPs selected for two production OPAs, 2279 were from ESTs and 793 from PCR amplicons. There was considerable intersection in the sets of SNPs provided by each identification path. For all OPAs preference was given to SNPs identified by amplicon sequencing. The final tally of surviving SNPs from each selection path included 65.9% (2279/3456) of the EST-derived and 69.6% (793/1140) of the PCR amplicon-derived SNPs. By this metric, the overall success rates were essentially equal for the two strategies for SNP discovery, ESTs versus genomic amplicon sequences.

The yield of SNPs from each of 253 pairwise genotype alignments of ESTs (see Supplemental Text for SNP selection details, Additional File 1) revealed a strong linear relationship (r^2 ^= 0.84) between the number of SNPs and the product of the number of ESTs. For example, the initial set of 36 pairwise genotype comparisons between eight malting barley cultivars and one wild barley accession (used for POPA1 and POPA2) is provided in Figure S1, Additional File 2), where this linear relationship and the higher frequency of SNPs when including the wild barley accession are readily apparent. In retrospect, it was fortuitous for SNP discovery that researchers in each country chose their own local favourite malting barley for EST sequencing.

### Genetic linkage maps

For each of the four mapping populations the linkage groups separated cleanly using MSTMap (see Methods) at LOD 4 or 5 and generally remained intact at higher LOD values. The four maps from individual crosses were fused using MergeMap (see Methods) to form a consensus map containing 2943 SNP loci with a total map length of 1099 cM (Table 1). The identity and polarity of linkage groups were determined by integrating 110 previously mapped bin markers [11] into the SxM and consensus maps (Table S1, Additional File 3). Because the SNP data are more complete and seem generally to be of higher quality than the SxM bin marker data, the 2943 "SNP-only" map and its distance coordinates are taken as the central point of reference in this paper (Figure S2, Additional File 4). Table S1 (Additional File 3) provides map coordinates for each of the four individual maps, the SxM map with 110 bin markers, the 2943 SNP-only consensus map and the 3053 marker consensus map containing 2943 SNPs and 110 SxM bin markers. The number and distribution of loci for each individual SNP-only map and the consensus SNP-only map are given in Table 1. In all maps, chromosome 5H has the greatest length, a mean of 198 cM, consistent with previously published linkage maps. Chromosome 5H is also the most populated with 535 SNP loci and is subdivided into the largest number of marker bins (180). On the lower end of the spectrum chromosome 4H has only 338 SNP loci distributed among 113 marker bins covering 125 cM. The relationship of nearly one marker bin per cM holds for all seven linkage groups.

**Table 1:** Distribution of SNPs in four individual maps and consensus map

		Chromosome							
**Map**	**Count type**	**1H**	**2H**	**3H**	**4H**	**5H**	**6H**	**7H**	**All**

**Morex × Barke**	**markers**	215	279	246	141	299	219	248	1652
	
	**bins**	60	72	77	39	74	54	65	443
	
	**cM**	134.0	151.9	178.1	112.4	195.7	133.8	158.9	1064.9

**Oregon Wolfe Barley**	**markers**	168	235	255	211	278	202	213	1562
	
	**bins**	65	73	91	60	89	64	67	509
	
	**cM**	145.4	181.0	199.3	121.8	231.1	152.3	186.7	1217.6

**Steptoe × Morex**	**markers**	148	217	242	130	225	122	183	1270
	
	**bins**	49	57	63	49	80	40	57	396
	
	**cM**	139.7	148.8	154.7	141.5	187.3	123.8	140.8	1036.6

**Haruna Nijo × OHU602**	**markers**	93	131	123	97	108	92	88	732
	
	**bins**	46	65	58	48	58	40	47	362
	
	**cM**	145.2	162.6	162.7	124.5	176.4	123.0	182.5	1076.7

**Consensus**	**markers**	341	485	475	338	535	352	417	2943
	
	**bins**	125	161	152	113	180	111	133	975
	
	**cM**	141.1	161.1	173.7	125.1	197.6	133.2	167.2	1099.0

**Figure 2:**
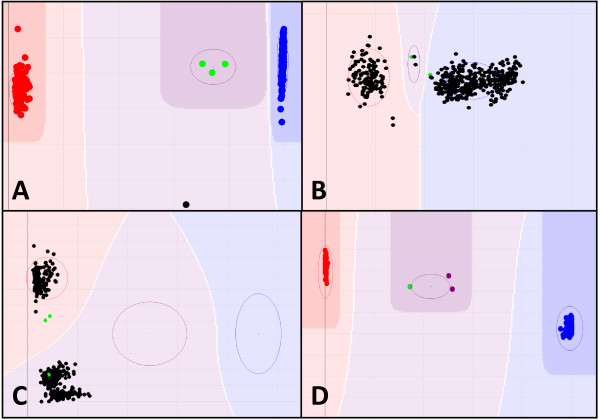
**Examples of SNP data**. A) Typical clustering of satisfactory data for POPA SNP 3_0004; red cluster area = homozygous AA, blue = homozygous BB, green dots within purple cluster area are 1:1 mixtures of parental DNA for three DH mapping populations. One germplasm sample (black dot) was outside of any call cluster and was thus scored "no call". B) Typical theta compressed data for POPA SNP 3_1104; although the polymorphism can be mapped in an individual population there are often wrong calls in such data and the cluster separation is problematic for general use in germplasm analyses or with multiple mapping populations; set to Gentrain 0.000, 100% "no call". C) Typical vertically separated clusters for POPA SNP 3_0070; generally polymorphic for a different locus than the source of the targeted SNP, which results in wrong annotation and degraded synteny; set to Gentrain 0.000, 100% "no call". D) Data for POPA SNP 1_1166 (ABC07305-1-4-322) from the OWB population; two DH samples behave as heterozygotes (purple cluster), far from the homozygotes (red = AA; blue = BB), instead with the 1:1 mixture of parental DNAs (green dot in purple cluster).

Once the SNP loci were arranged by position on the consensus map, graphical visualization enabled inspection of the distribution of recombination events. The genotype data and graphical genotype displays for three of the four mapping populations (MxB, OWB, SxM) are provided in Table S2 (Additional File 5), where it can be seen that there are no singleton double recombinant loci in densely marked regions of any of the maps. Since such loci are often indicative of genotyping errors, the complete absence of suspicious double recombinants can be considered an indicator of high fidelity of the data from the 2943 SNP loci selected for linkage map production. Other quality metrics include the frequency of missing data or apparent heterozygosity; aside from two instances of apparent heterozygosity at locus 1_1166 in two seemingly identical OWB doubled haploid lines #22 and #70 (Figure 2D, Table S2, Additional File 5), all individuals in all three mapping populations had homozygous genotype calls for all loci and no missing data. This is 100% of 153,636 possible genotype calls in the MxB population, 99.999% of 145,266 possible genotype calls in the OWB population and 100% of 116,840 possible genotype calls in the SxM population. The high fidelity and lack of missing data among these selected 2943 SNPs facilitated the production of individual and consensus maps. More than 300 SNPs with imperfect but still high quality data (for example 3_1104, Figure 2C) were not utilized for this map.

**Figure 3:**
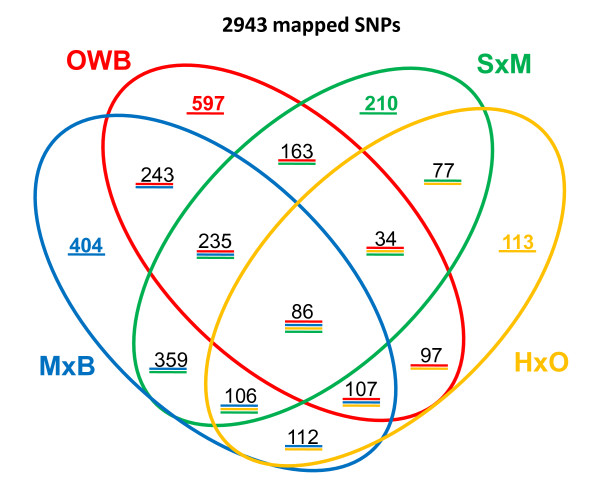
**Venn diagram showing marker overlap**. A four-way Venn diagram illustrates all unique, two-way, three-way and four-way sets of shared markers. The mapping populations are abbreviated as in the text: MxB = Morex × Barke, OWB = Oregon Wolfe Barley, SxM = Steptoe × Morex, HxO = Haruna Nijo × OHU602.

**Figure 4:**
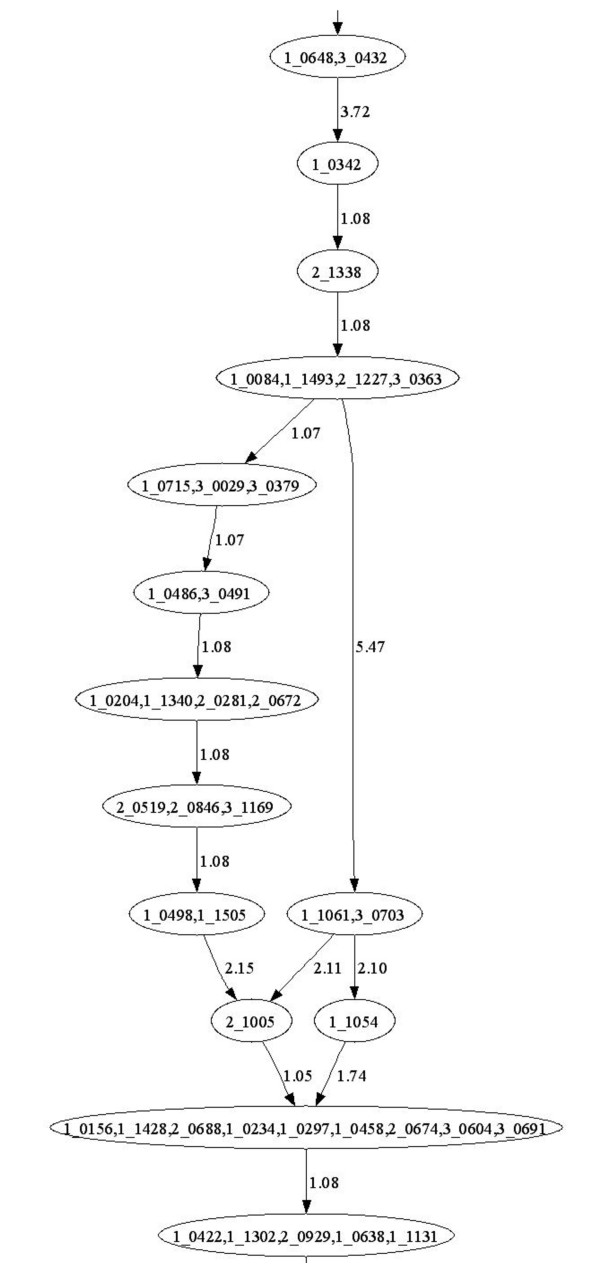
**Segment of a consensus directed acyclic graph**. A typical segment of a directed acyclic graph representing the consensus map of one barley linkage group is shown. Each oval represents one bin of SNP markers, using POPA names for SNPs. Where an oval contains more than one SNP, it means that there was no evidence of recombination in any mapping population between those markers. The observed recombination frequencies between marker bins are shown. The exact order of marker bins cannot be solved with certainty unless markers are shared between maps. Recombination frequencies are often not proportional to physical distance, nor consistent, when comparing two or more maps from different mapping populations. Therefore directed acyclic graphs provide a more exact description of the limit of knowledge of the marker order than does a linear map derived using approximations based on recombination values. See the text for further discussion.

Figure 3 illustrates the number of shared markers between any two, any three and all four maps. The substantial number of shared markers facilitated the production of a consensus map. The number of pairwise shared markers ranged from 303 between the HxO and OWB maps to 786 shared between the MxB and SxM maps. Three-way shared markers range from 120 when including all maps except MxB to 321 when including all maps except HxO. The lower number of shared markers involving the HxO map is due to the fact that this population was genotyped using only BOPA1, whereas the other three populations were genotyped using all three Pilot OPAs (see Methods). Table S1 (Additional File 3) provides complete information on the map locations of all markers, where it can also be seen that there was no disagreement in the order of shared markers in any of the six pairwise comparisons of linkage maps, or between the consensus map and any individual map. It should be noted, however, that this does not guarantee that the marker order in the 2943-SNP consensus map perfectly matches the order of the corresponding nucleotides within the genome sequence. The consensus map is simply one of many possible non-conflicting linear representations of the consensus DAGs (Figure 4, Figures S3-S9, Additional Files 6, 7, 8, 9, 10, 11 and 12). The limit of knowledge of non-shared marker order is more accurately shown in the consensus DAGs of each linkage group. As more data accumulate from additional mapping populations, linkage disequilibrium analyses and genome sequencing, the number of non-conflicting linear map orders will be reduced, ideally to just one possible order. Naturally, the consensus map will evolve toward finer resolution and convergence on the correct order of all markers.

Segregation distortion was observed in all four mapping populations, being most pronounced in the MxB population in the pericentric regions of 1H, 2H, 5H and 7H and the long arm of 7H. Interestingly, on 7H the distortion was toward the maternal allele (Morex) in the pericentric region but toward the paternal allele (Barke) on the long arm.

### Alternative marker names

Table S3 (Additional File 13) provides a cross-reference between synonymous marker names, relating SNPs mapped in the present work to the same genes mapped previously using other marker systems [3]. To generate this cross reference, all of the HarvEST:Barley assembly #35 unigenes (U35; Table S4, Additional File 14) were searched using BLASTN against the GrainGenes "Sequenced Probes" database http://wheat.pw.usda.gov/GG2/index.shtml at a cut-off of 1e-10. Probes that matched more than three U35 unigenes were ignored. The result was 636 previously mapped probes matching 1114 unigenes. The list of probes was then reduced to those mapped in Marcel et al. [3] and the list of unigenes was limited to those which were sources of the 2943 mapped SNPs. Finally, the map position of the SNP and the previously mapped probe were compared, discarding a few with gross mismatches in genome location (presumably paralogous loci mapped by the probe). The resulting intersection contains 55 SNPs representing 51 U35 unigenes matching 51 sequenced probes. By comparing the map positions in Table S3 (Additional File 13) one can see that there is perfect co linearity of shared marker order between the two maps, though there are differences in map distances throughout each linkage group. There are no shared markers on 4HL, which illustrates the need for a more comprehensive cross-reference resource than just these two consensus maps. A similar operation can be performed to relate other maps to the present 2943 SNP map. It should be noted also that the original SNP names from SCRI ("ABC" format, Table S4, Additional File 14) correspond in many cases to SNPs used in Rostoks et al. [1] and the original SNP names from IPK ("ConsensusGBS" format, Table S4, Additional File 14) correspond to SNPs in Kota et al. [12]. Thus, those two maps can be readily cross-referenced to the present map using in-common marker names. Also, as stated above, 110 bin markers from the SxM map of Kleinhofs and Graner [11] are included in Table S1 (Additional File 3). Overall, cross-referencing the 2943 SNP-only map to previous maps provides an important bridge between additional resources including a physical map now being coupled to the 2943 SNP-only map and QTLs, simple trait determinants and deletion sites that already have been mapped in prior work. Szűcs et al. [10] included 1472 of the SNPs developed in the present work in addition to SSRs, AFLPs and DArT markers, making the resulting OWB map an excellent new point of cross-reference for barley markers.

### Synteny

**Figure 5:**
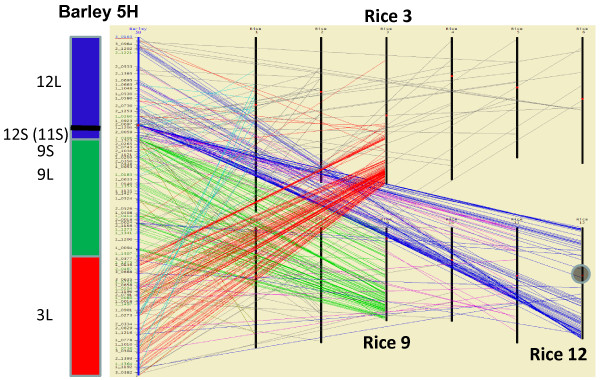
**Barley-rice synteny in detail for 5H**. HarvEST screenshot showing barley-rice synteny for chromosome 5H. Colored lines connect each barley locus to the position of the best BLAST hit on the rice genome.

**Figure 6:**
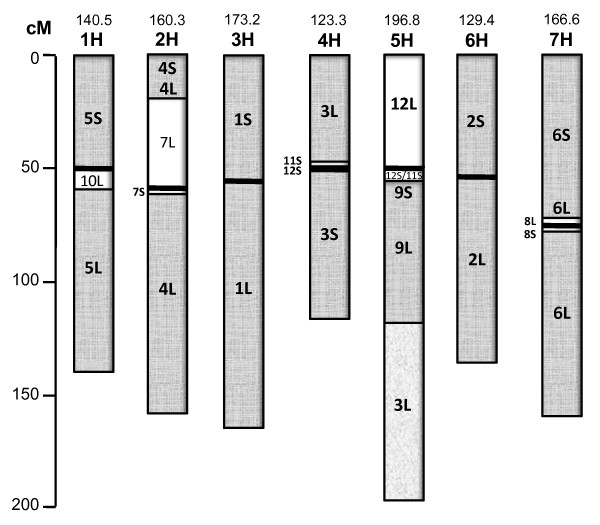
**Barley-rice synteny summary**. Seven barley linkage groups represented as rice synteny blocks. Numbers inside each barley chromosome indicate syntenic rice chromosome arm.

Each barley SNP source sequence was compared to the rice (*Oryza sativa*) version 5 and version 6 gene models [13] using BLASTX, and the top hit was taken as the most similar rice gene. These rice best hit coordinates were used as the basis of alignments of each of the seven barley chromosomes with the twelve rice chromosomes. Figure 5 is a screen shot from HarvEST:Barley [14] showing a detailed alignment of barley chromosome 5H with rice chromosomes. From this and each of the other six barley-rice alignments the marker density is sufficient to clearly reveal major elements of barley-rice synteny, consistent in general with prior publications on Triticeae-rice synteny (for example [15,16]. The short arm of barley 5H is syntenic with rice 12 L. The long arm of barley 5H is syntenic with an interspersion of rice 12S and 11S genes followed by rice 9S, then rice 9 L, then rice 3 L. The position of the centromere in each barley chromosome was determined using flow-sorted chromosome arms in work that will be described in detail elsewhere (Prasanna Bhat et al. in preparation). Of the seven barley chromosomes, 5H has the most complex barley-rice synteny relationship, being the only barley chromosome composed of major syntenous blocks from more than two ancestors of rice chromosomes. An illustration of barley-rice synteny for all seven barley chromosomes is provided in Figure 6. The simplest relationships are essentially total synteny between barley 3H versus rice 1 (3HS = 1S, 3HL = 1L) and barley 6H versus rice 2 (6HS = 2S, 6HL = 2L). The four remaining barley chromosomes each are composed of ancestors of two rice chromosomes, in each case having one ancestral chromosome nested within the pericentric region, flanked by segments of the other syntenic chromosome. Detailed views of synteny similar to Figure 5, but with zoom-in and active links to external databases, are available for all seven chromosomes through the Windows version of HarvEST:Barley [14].

An interesting consequence of the evolutionary history of barley chromosomes is that the number of expressed genes in the pericentric regions is highly variable, ranging from relatively few in the cases of barley 3H and 6H to very many in the most extreme case of barley 7H. The relative genetic map density of expressed genes has major implications for plant breeding efforts. If, for example, a trait maps to an ancestral telomeric region within the pericentric region of barley 7H then it will be much less likely that the gene controlling that trait can be separated from neighbouring genes by recombination than, for example, a gene located in the ancestral centromeric region of rice chromosome 9, which is located in a more recombinationally active region on barley chromosome 5H. For example there have been several as yet unsuccessful attempts to map-base clone *Mlg*, a powdery mildew resistance gene located in the gene-dense pericentric region of 4H (Matthew Moscou, unpublished data). Similarly, the level of difficulty in map-based cloning efforts will also depend on the gene density in regions of low recombination. Due to high gene density in ancestral telomeric regions which are now nested within barley chromosomes, the pericentric regions of five barley chromosomes (1H, 2H, 4H, 5H and 7H) have high gene density.

The version 6 rice genome sequence coordinates, including chromosome, arm and base pair position, are included in Table S4 (Additional File 14), along with the chromosome and arm position from version 5. The 2943 genetically mapped barley SNPs were derived from 2786 source sequences, of which 2703 have a rice BLASTX match of at least 7 × e^-5^. A total of 36 of these had a best BLASTX against a gene positioned to different rice chromosomes when comparing ortholog locations in rice version 5 to version 6. It is interesting that 14 of the 36 (39%) changes in the rice genome annotations brought rice-barley synteny into line with the barley genetic map, 9 of the 36 (25%) changes degraded rice-barley synteny and 12 (33%) had a neutral effect because neither the version 5 nor 6 rice annotations were syntenic with barley. One had a neutral effect because both BLAST hits were consistent with syntenic duplications in the barley and rice genomes. It is not unusual to find imperfect synteny such as the 12 of 36 (33%) revised but non-syntenic positions; in fact 745 of the 2703 mapped barley SNP source sequences (27.6%) do not point to a best rice BLASTX within the major synteny block. However, from this comparison of the edited positions in rice versions 5 versus 6 to the 2943 SNP barley genetic linkage map, it appears that the barley SNP map is the more stable point of reference. Consequently, it may be of some benefit to use the barley genetic map for further revisions of the rice genome sequence.

### BOPA1 and BOPA2 elements and performance

As discussed above, the two production OPAs, BOPA1 and BOPA2, had somewhat different design elements. These differences have been reflected in the performance of BOPA1 and BOPA2 for the genotyping of breeding germplasm within the BarleyCAP project [17]. Table 2 provides a comparison of BOPA1 and BOPA2 in relation to both SNP representation and the performance on 960 year 2006 US breeder DNA samples in the BarleyCAP project. Table 2 also summarizes information provided in greater detail in Table S4 (Additional File 14) on the relationship of BOPA SNPs to probe sets on the Affymetrix Barley1 GeneChip [18] and the 2943 mapped SNPs in the present work. Extensive analyses of the diversity of breeding germplasm will be the subject of other papers; here we emphasize only the elements and fundamental performance characteristics of BOPA1 and BOPA2. One can see that BOPA1, which was designed using only SNPs with a minor allele frequency (MAF) of at least 0.08 in the design germplasm, yielded MAF values less than 0.05 for only 164 SNPs (10.7%) in the US breeding materials. In contrast BOPA2, which targeted 615 SNPs with MAF less than 0.08 in the design germplasm, yielded MAF values less than 0.05 for 585 SNPs (38.1%) in the breeding germplasm. This included about three times as many SNPs with MAF = 0 (301 versus 99) and 4.4 times as many SNPs (284/65) with MAF between 0 and 0.05. Thus, BOPA2 has greater sensitivity to detect rare alleles than does BOPA1, some of which may be important for the development of new varieties containing uncommon alleles of certain genes. But, this increased sensitivity is counterbalanced by a compromise in the reduced frequency of informative SNPs in general.

**Table 2:** Design and performance characteristics of BOPA1 and BOPA2

	BOPA1	BOPA2	Both
**SNPs represented**	1536	1536	3072

Number of unigenes on other BOPA*	77	77	NA

**Unigenes represented**	1536	1442	2901

**Number of unigenes with 1 SNP**	1536	1380	2770

**Number of unigenes with 2 SNPs**	0	43	106

**Number of unigenes with 3 SNPs**	0	11	16

**Number of unigenes with 4 SNPs**	0	3	3

**Number of unigenes with 5 SNPs**	0	5	6

**SNPs included in 2943 mapped**	1414	1263	2677

**SNP unigenes matching Barley1 probe set(s)**	1489	1433	2921

**MAF ≥ 0.08 in design germplasm**	1536	921	2457

**MAF ≥ 0.04 and < 0.08 in design germplasm**	0	256	256

**MAF ≥ 0.005 and < 0.04 in design germplasm**	0	345	345

**MAF = 0 in design germplasm**	0	14	14

**MAF = 0 in 2006 BarleyCAP genotypes**	99	301	400

**MAF > 0 and < 0.05 in 2006 BarleyCAP genotypes**	65	284	349

**MAF ≥ 0.05 in 2006 BarleyCAP genotypes**	1372	951	2323

*Among the 77 unigenes represented by SNPs on both BOPAs, 69 have 1 SNP on BOPA2, 6 have 2 SNPs on BOPA2, 1 has three SNPs on BOPA2, 1 has four SNPs on BOPA2.

Table S5 (Additional File 15) lists the MAF values determined during the design of BOPA1 and BOPA2 versus the observed MAF values in year 2006 and year 2007 BarleyCAP breeding germplasm. For example, 11 of 157 SNPs with a design MAF of 0.01 or lower had an observed MAF of at least 0.08 in year 2006 or 2007 breeding germplasm. Similarly, 25 of 283 SNPs with a design MAF of 0.024 or lower had an observed MAF of at least 0.10 in year 2006 or 2007 breeding germplasm. The differences between BOPA1 and BOPA2 should be carefully considered by potential users, and the characteristics of specific SNPs should be considered when selecting subsets of SNPs for other platforms.

### Other characteristics of the 2943 SNP map

It is perhaps of relevance that there were significant differences in the genetic length of some of the individual chromosomes in the different populations (Table 1). For example, the genetic length of chromosome 4H in the SxM population is expanded relative to the same chromosome in any other population, and all of the other chromosomes have a longer genetic length in the OWB population than in the other populations. Also, the genetic map lengths are consistently higher than would be expected from cytogenetic counts of chiasmata per meiosis for this species, as previously noted [19] despite the fact that methodological errors in genotyping can be ruled out in the present work because of the lack of any suspicious-looking singleton double recombinants. The notable deviations from mean genetic distance values indicate that the genetic background as well as environmental factors may have had a significant effect on recombination, and presumably also chiasmata counts, in this species. Also, although the broad patterns of synteny within grasses recognized previously by many investigators has been confirmed by this work, the hitherto unprecedented density of gene-derived markers enable further delineation of several inversions and rearrangements of gene order at macro-, meso- and micro-synteny levels. Chromosome 5H (Figure 5) provides one example of such rearrangements at the macrosynteny level. The HarvEST:Barley http://harvest.ucr.edu synteny viewer provides zoom-in functionality to enable visualization at meso and micro-synteny levels as well.

### Access to the linkage map and SNP data

The 2943 SNP linkage map can be accessed by several browsers including HarvEST:Barley [14] or [20], GrainGenes [21], NCBI [22] and THT [23]. New versions of the map may become available as additional mapping populations are applied to BOPA1 and BOPA2, linkage disequilibrium is used for mapping and the physical map and genome sequence are coupled to the genetic linkage map.

## Conclusion

The unprecedented density of genic markers and marker bins enabled a high resolution comparison of the genomes of barley and rice. Low recombination in pericentric regions is evident from bins containing many more than the average number of markers, meaning that a large number of genes are recombinationally locked into the genetic centromeric regions of several barley chromosomes. Examination of US breeding germplasm illustrated the usefulness of BOPA1 and BOPA2 in that they provide excellent marker density and sensitivity for detection of minor alleles in this genetically narrow material.

## Methods

### Five 1536-SNP GoldenGate assays (Figure 1, Table 2)

Three pilot-phase 1536-SNP GoldenGate assays were developed. These "pilot OPAs" are referred to as POPA1, POPA2 and POPA3. Two 1536-SNP production-scale OPAs, referred to as BOPA1 and BOPA2, were developed from SNPs tested on the pilot OPAs. All sequences used as SNP sources were generated using the Sanger dideoxy chain termination method.

### POPA1 and POPA2

The contents of POPA1 and POPA2 came from an initial list of SNPs comprised of the union of three intersecting lists from SCRI (1,658 SNPs), IPK (985 SNPs) and UCR (12,615 SNPs). SCRI and IPK SNPs were derived from PCR amplicon sequences, whereas UCR SNPs were derived nearly entirely from EST sequences. In the selection of SNPs for the OPAs, preference was given to SNPs derived from amplicon sequences. Nearly all SNPs on POPA1 and about 60% of the SNPs on POPA2 targeted stress-regulated genes. The composition of POPA1 included 1524 barley SNPs, one per gene, of which 1033 were derived from ESTs and 491 from amplicon sequences. The composition of POPA2 included 1536 barley SNPs, one per gene including 258 genes represented on POPA1, of which 1456 were from ESTs and 80 from amplicon sequences.

### BOPA1

BOPA1 represented 705 SNPs from POPA1 and 832 from POPA2, including one SNP in common. All BOPA1 SNPs had a satisfactory technical performance on POPA1 or POPA2 and a minor allele frequency of at least 0.08. To the extent of results presented in this manuscript, BOPA1 included 1414 mapped and 122 unmapped SNPs.

### POPA3

Residual SNPs from the sources of POPA1 and POPA2 were insufficient to complete the design of POPA3 without compromising on the SNP selection criteria. Additional SNPs for POPA3 came from three sources: 1) an extended list of 5,732 SNPs identified in SCRI amplicon sequences, 2) colleagues who contributed SNPs from amplicon sequences of specific genes of biological interest and 3) an expanded barley EST resource. The first two of these additional sources were exhausted for POPA3 design. In the selection of EST-derived SNPs, priority was given to genes previously classified as having interesting expression patterns during malting or upon exposure to pathogens, or relevant to malting, brewing quality, abiotic stress or phenology. The composition of POPA3 included 1536 barley SNPs, in many cases more than one per gene and in some cases including genes represented on POPA1 or POPA2. In total, 967 POPA3 SNPs were derived from ESTs and 569 from amplicon sequences.

### BOPA2

BOPA2 represented 406 SNPs from POPA1, 178 from POPA2 and 952 from POPA3. The primary emphases of BOPA2 were representation of mapped SNPs that were not included on BOPA1 and inclusion of multiple SNPs for certain genes to reveal haplotypes at these loci, with some weight given to MAF. BOPA2 contained 921 SNPs with MAF at least 0.08, 256 SNPs with MAF at least 0.04 but less than 0.08, 345 SNPs with MAF least 0.005 but less than 0.04, and 14 SNPs with only one allele (MAF = 0) in the germplasm examined using POPA3. To the extent of results presented in this manuscript, BOPA2 included 1263 mapped and 273 unmapped SNPs. A total of 967 SNPs were from ESTs and 569 from amplicon sequences.

### SNP annotations

Table S4 (Additional File 14) provides alternative SNP names arising from this work, and several annotation fields for all SNPs represented on POPA1, POPA2, POPA3, BOPA1 and BOPA2. The annotations include BLAST hits to the rice and Arabidopsis genomes and UniProt, the relationship of SNP source sequences to HarvEST:Barley unigenes and probe sets on the Affymetrix Barley1 GeneChip and source consensus sequences. To assign SNP loci on the genetic map to chromosome arms, centromere positions were identified using flow-sorted chromosome arms following the method described in Simkova et al. [24]; results of this work will be described elsewhere (Bhat et al., in preparation). The annotation information in Table S4 (Additional File 14) is also available from HarvEST:Barley [14] and [20]. The HarvEST BLAST server [25] provides the 2943 mapped SNP unigene sequences as a searchable database.

### DNA sources

Genomic DNAs of 93 doubled haploid maplines and the parents (Dom, Rec) of the Oregon Wolfe Barley (OWB) population [26,27] 148 doubled haploids and the parents of the Steptoe × Morex (SxM) population [7,28], 95 doubled haploid maplines and the parents of the Haruna Nijo × OHU602 (HxO) population and 213 additional germplasm samples were purified using Plant DNeasy (Qiagen, Valencia, CA, USA) starting with 100-300 mg of young seedling leaves. Genomic DNAs of 93 doubled haploid maplines and the Barke parent from the Morex × Barke population (Stein et al. unpublished) were produced using a CTAB method. All DNA samples were checked for concentration using UV spectroscopy and Quant-iT PicoGreen (Invitrogen, Carlsbad, CA, USA) and adjusted to approximately 120 ng/μl in TE buffer.

### Data production for map construction and MAF estimation

DNA concentrations were re-checked using Quant-iT PicoGreen (Invitrogen, Carlsbad, CA) and standardized to 80 ng/μl in TE buffer in preparation for the GoldenGate assay and 5 μl (400 ng) were used for each assay. Data were generated from each progeny line in the OWB, SxM and MxB doubled haploid populations using POPA1 and POPA2. Data were also produced using POPA3 from the complete OWB and MxB sets of DNA samples, but from only 92 SxM doubled haploids. Data from 95 HxO doubled haploids using BOPA1 were also generated. For each of these four mapping populations, extensive integration of SNP data with other types of marker data will be described elsewhere (for example OWB marker integration in Szűcs et al. [10]). Data used for the determination of allele frequency (see below) came from 125 germplasm samples for POPA1, 195 germplasm samples for POPA2, and 189 germplasm samples for POPA3.

### Data processing

Raw data were transformed to genotype calls, initially using Illumina GenCall and subsequently using Illumina BeadStudio version 3 with the genotyping module. For each OPA, the data from all samples were visually inspected to manually set 1536 archetypal clustering patterns. The cluster positioning was guided by knowledge that heterozygotes are nearly non-existent in doubled haploids and rare in highly inbred parental genotypes and germplasm samples. Several "synthetic heterozygote" DNA samples were made by mixing parental DNAs in a 1:1 mass ratio (Figure 2A, green dots), and included to anchor heterozygote cluster positions to enable the identification of true heterozygotes which occur at a significant frequency in germplasm samples that have not been sufficiently inbred to reach a state of genome-wide allele fixation. The spatial positions of heterozygote and homozygote data clusters were confined to areas of high certainty so that data points with less certainty fell outside the boundaries of heterozygotes and homozygotes and were scored as "no-call" (Fig 2A, one germplasm sample as black dot). Polymorphisms with theta compressed clusters were not used if the compression was such that any homozygote call was not plainly distinguishable (Figure 2B, set as Gentrain 0.000, 100% "no call"). Vertically separated data clusters were not accepted as polymorphisms (Figure 2C). Following the production of one master workspace for each Pilot OPA using all DNA samples, customized workspaces were produced for each mapping population to optimize the genotype calls via minor adjustments of cluster positions. Genotype calls were exported as spreadsheets from BeadStudio and then parsed to create input for mapping programs.

### Individual and consensus map production

Individual maps were made principally using MSTMap [29,30] for each data set from the four doubled haploid mapping populations. In brief, MSTMap first identifies linkage groups, then determines marker order by finding the minimum spanning tree of a graph for each linkage group, then calculates distances between marker using recombination frequencies. JoinMap 4 [31] was used to confirm linkage groups and marker order determined by MSTMap. Raw data for problematic markers were reviewed using BeadStudio and then either the marker was discarded entirely if any ambiguity in data calling could not be resolved or individual genotype calls were modified if it was plainly evident that such adjustments were warranted. Each such review of primary data was followed by the production of new maps; this iterative process generally involved 10-20 cycles for each individual map. At several points, a consensus map was produced using MergeMap [32], which also flags problematic markers for review. MergeMap takes into account marker order from individual maps and calculates a consensus marker order. Briefly, the input to MergeMap is a set of directed acyclic graphs (DAGs) [33] from each individual map, and the output is a set of consensus DAGs (Figure 3, Figures S3-S9, Additional Files 6, 7, 8, 9, 10, 11, 12), where each is consistent with all (or nearly all) of the markers in the individual input maps. MergeMap then linearizes each consensus DAG using a mean distance approximation. The consensus map coordinates from MergeMap were normalized to the arithmetic mean cM distance for each linkage group from the four individual maps (Figure S2, see Additional File 4 and Table S4, see Additional File 14).

### Implementation of BOPA1 and BOPA2 in US barley breeding germplasm

As part of Barley CAP [17], the two BOPAs have been part of an effort to genotype a total of 3840 US barley breeding lines contributed from ten US barley breeding programs for association mapping analyses. As of January 2009, data from both BOPAs had been generated for 1920 breeding lines, with 960 submitted from the selections of each of two years, 2006 and 2007. Table S5 (Additional File 15) provides MAF for observed in these samples for each SNP in BOPA1 and BOPA2.

## Abbreviations

AFLP: amplified fragment length polymorphism; DAG: directed acyclic graph; DArT: diversity array technology; EST: expressed sequence tag; QTL: quantitative trait locus; RFLP: restriction fragment length polymorphism; SNP: single nucleotide polymorphism; SSR: simple sequence repeat; STS: sequence tagged site.

## Authors' contributions

The contributions of authors and other colleagues, locations of work conducted and cost sharing are detailed in Supplemental Text (Additional File 1), which contains citations of references 34-39 and Additional Files 16, 17, 18, 19, 20 and is essentially an expanded version of Methods.

## Supplementary Material

Additional file 1 Supplemental Text Click here for file

Additional file 2 **Figure S1**. SNP yield. The near-linear relationship between the number of SNPs and the product of the number of EST sequences for pairwise genotype comparisons is shown by plotting all values versus a linear regression line. Each axis is on a logarithmic scale. Oval shapes indicate a comparison involving the wild barley accession OHU602. See text for additional details. Click here for file

Additional file 3 **Table S1**. All individual and consensus maps, including SxM bin markers. Click here for file

Additional file 4 **Figure S2**. Consensus 2943 SNP genetic linkage map. Click here for file

Additional file 5 **Table S2**. All data from MxB, OWB and SxM mapping populations. Click here for file

Additional file 6 **Figure S3**. Complete consensus directed acyclic graphs for barley chromosomes 1H. Click here for file

Additional file 7 **Figure S4**. Complete consensus directed acyclic graphs for barley chromosomes 2H. Click here for file

Additional file 8 **Figure S5**. Complete consensus directed acyclic graphs for barley chromosomes 3H. Click here for file

Additional file 9 **Figure S6**. Complete consensus directed acyclic graphs for barley chromosomes 4H. Click here for file

Additional file 10 **Figure S7**. Complete consensus directed acyclic graphs for barley chromosomes 5H. Click here for file

Additional file 11 **Figure S8**. Complete consensus directed acyclic graphs for barley chromosomes 6H. Click here for file

Additional file 12 **Figure S9**. Complete consensus directed acyclic graphs for barley chromosomes 7H. Click here for file

Additional file 13 **Table S3**. Synonymous marker names. Click here for file

Additional file 14 **Table S4**. All marker consensus map coordinates, names, source types, BLASTs, probe sets, sequences. Click here for file

Additional file 15 **Table S5**. Minor allele frequencies for each SNP on BOPA1 and BOPA2. Click here for file

Additional file 16 **Table S6**. POPA1 SNPs. Click here for file

Additional file 17 **Table S7**. POPA2 SNPs. Click here for file

Additional file 18 **Table S8**. POPA3 SNPs. Click here for file

Additional file 19 **Table S9**. BOPA1 SNPs. Click here for file

Additional file 20 **Table S10**. BOPA2 SNPs. Click here for file
