# Supplementary material for: Development and implementation of high-throughput SNP genotyping in barley
Source: BMC Genomics. 2009 Dec 4;10:582. doi: 10.1186/1471-2164-10-582 (PMC2797026; doi:10.1186/1471-2164-10-582)
Supplement: Additional file 1 — Supplemental Text [file 1471-2164-10-582-S1.DOC]

**Methods**

**Five 1536-SNP GoldenGate assays (Fig. 1)**

Three pilot-phase 1536-SNP GoldenGate assays were developed. Oligonucleotides were produced at a quantity sufficient to genotype 480 DNA samples using standard procedures available from Illumina (San Diego, CA, USA). These “pilot OPAs” are referred to as POPA1, POPA2 and POPA3. Remnants were used to extend the POPA1 and POPA2 material to 96 additional DNA samples. Two 1536-SNP production-scale OPAs, referred to as BOPA1 and BOPA2, were developed from SNPs tested on these pilot OPAs. The 4608 oligonucleotide sequences for each 1536-plex assay are provided in Illumina “manifest file” format in Tables S6-S10 (Additional Files 16 – 20).

POPA1 was designed November 2004 to May 2005 (TJC, SW, JTS) to represent 1524 barley and 12 citrus SNPs and purchased jointly by UCR (TJC), SCRI (RW) and IPK (NS, AG). Every other OPA represented 1536 barley SNPs. POPA2 was designed January to March 2006 (TJC, LR, SW, KM) and purchased by UCR (TC). POPA3 was designed from March 2006 through October 2007 (TJC, NR, SW) and purchased by SCRI (RW). BOPA1 was designed December 2006 to January 2007 (TJC), purchased jointly by U Minnesota (GJM) and SCRI (RW). Sufficient BOPA1 was produced for 20,064 DNA samples. BOPA2 was designed from January to March 2008 (TJC, PB, DFM) and purchased by U Minnesota (GJM and Kevin Smith), Oregon State University (Patrick Hayes) and UCR (TJC). Sufficient BOPA2 was produced to genotype 10,080 DNA samples.

**SNPs**

All EST and PCR amplicon sequences were generated using the Sanger dideoxy chain termination method.

**POPA1, POPA2, BOPA1 SNP Sources**

The contents of POPA1 and POPA2, and therefore BOPA1 (derived entirely from POPA1 and POPA2), came from the union of three intersecting SNP lists from SCRI (NR), IPK (NS, RV) and UCR (TJC, SW). A list of 1658 SNPs in 572 contigs from SCRI was developed by alignment of sequences from PCR amplicons derived from eight barley genotypes (Oregon Wolfe Barley Dom, Oregon Wolfe Barley Rec, Steptoe, Morex, Lina, HS92, Golden Promise, Optic). As described in Rostoks et al. [1], most of these SNPs were in abiotic stress-regulated genes. Several stress-regulated lists used for gene selection in Rostoks et al. [1], and for categorization of SNPs in the UCR list (below), were from Walia et al. [34] (salinity), Svensson et al. [35] (chloroplast-dependent low temperature) and Tommasini et al. [36] (drought and low temperature). The name convention for SCRI SNPs begins with “ABC”, followed by the HarvEST:Barley [14, 20] assembly #21 unigene number, followed by the SNP position. For example, ABC07525-1-1-70 is a SNP at position 70 in the PCR amplicon sequence corresponding to HarvEST:Barley assembly 21 unigene number 7525. Assembly #21 was the basis of the Affymetrix Barley1 GeneChip [18]. Another 985 SNPs in 220 contigs from IPK were derived by alignment of PCR amplicons from seven genotypes (Igri, Franka, Steptoe, Morex, Oregon Wolfe Barley Dom, Oregon Wolfe Barley Rec, Barke), as described in Kota et al. [12]. The name convention for IPK SNPs begins with “ConsensusGBS”, followed by the contig number of aligned amplicon sequences. A total of 12,615 SNPs in 3509 contigs from UCR were identified by alignment of EST sequences in assembly #32 of the HarvEST:Barley database [14,20]. The name convention for UCR SNPs is the assembly #32 unigene number followed by the position of the SNP. For example, 3897-578 is a SNP at position 578 in the consensus sequence of assembly #32 unigene 3897. Assembly #32 contained 351,645 sequences from 267,439 clones and was made using CAP3 [37] with parameter settings p = 75, d = 240, f = 250, h = 90. These relatively relaxed settings result in alleles being combined into a single contig more often than with relatively stringent settings of p = 95, d = 60, f = 100, h = 50 which were used for assembly #21. SNPs were identified from 36 pairwise comparisons of ESTs from eight malting barley cultivars (Akashinriki, Barke, Golden Promise, Haruna Nijo, Kymppi, Morex, Optic, Saana) and one wild barley accession (OUH602). Principal contributors of these ESTs were authors KS, NS, RW and DFM, and colleagues Rod Wing and Dorrie Main (Clemson University Genomics Institute) and Alan Schulman (University of Helsinki). For pairwise genotype comparisons, a SNP was accepted only if there were at least two sequences from each genotype and no disagreement at the SNP position between opposite strands from a single clone. A base call was used only if its Phred quality value was at least 25 and its position was at least 25 bases from the end of an EST sequence and not inside a window of 5 bases containing three or more Phred values less than 25. A total of 57 predicted SNPs were tested by amplicon sequencing (PC, RDF, JS, TJC), yielding 52 (91%) validated SNPs. In addition, within-Morex and within-Barke SNPs were accepted if there were at least three examples of each sequence and all other constraints were satisfied. A SNP was eliminated from further consideration if it was within 30 bases of an intron (deduced by alignment with the TIGR rice genome sequence version 3.0) or the end of the unigene sequence. This reduced the UCR list to 10,956 SNPs in 3353 contigs. HarvEST:Barley is a FoxPro database, so the SNP finding algorithm was implemented in the context of the FoxPro programming environment (SW). The compilation of 13,599 remaining SNPs described above was further reduced to 9180 SNPs by eliminating those with an Illumina SNP design score less than 0.59. This was 7549 UCR SNPs from 3055 assembly #32 unigenes, 1072 SCRI SNPs from 516 contigs, and 557 IPK SNPs from 204 contigs. These remaining SNPs were prioritized for representation on POPA1 or POPA2, as described below; POPA1 and POPA2 each contained one SNP per targeted gene.

**POPA1 SNP Selection**

Relationships between SCRI or IPK contigs and HarvEST:Barley assembly #32 unigenes were determined by finding within assembly #32 unigenes ESTs that were the origination points for SCRI and IPK amplicon sequencing, or in a few cases using BLAST to find a strong match to the amplicon consensus sequence. Assembly #32 unigenes corresponding to SCRI SNPs were given first priority for representation on POPA1. Assembly #32 unigenes corresponding to IPK SNPs were given second priority. The entire union of these two intersecting sets of assembly #32 unigenes was represented on POPA1, ultimately leading to 642 of the final 1524 barley SNPs; the remaining 882 assembly #32 unigenes represented on POPA1 depended entirely on SNPs from the UCR list. Several prioritization steps were then applied since there were excess UCR SNPs available to fill POPA1. Abiotic stress gene lists derived from experiments conducted using the Affymetrix Barley1 GeneChip (for example [34-36]) were related to assembly #32 unigenes to mark a portion of UCR SNP-bearing unigenes as “stress regulated”, and these were given third priority. Fourth priority was given to UCR SNP-bearing unigenes associated with single feature polymorphisms using stress-induced RNA as a genotyping probe or validated by amplicon sequencing [38]. UCR SNPs supported by only one pairwise genotype comparison were then eliminated, except when the genotype pair was Morex/Barke. The number of instances of each UCR SNP in 36 pairwise genotype comparisons was tallied and a weight factor was then added to the Illumina SNP score to bias UCR SNP selection in favor of SNPs with high MAF. For assembly #32 unigenes corresponding to SCRI or IPK SNPs, the SNP with the highest overall SNP score from any of the three SNP source paths was selected to represent that unigene. This means that, at this point, some UCR and IPK SNPs trumped SCRI SNPs, and some UCR SNPs trumped IPK SNPs. After all of the above steps a total of 1827 assembly #32 unigenes were still under consideration. BLAST hits against rice (TIGR version 3) gene models were examined to condense the list to only one case of each rice gene model, retaining the SNP with the best SNP score and its assembly #32 unigene number. This reduced the number of assembly #32 unigenes under consideration to 1662. Finally, UCR SNPs with the lowest SNP score were eliminated among SNPs which had been included only by being categorized as stress-related. The final composition of POPA1 included 1524 barley SNPs originating from 1033 UCR, 380 SCRI and 111 IPK SNPs. The original SNP names and a simplified naming convention, 1_0001 through 1_1536, are included in Table S4 (Additional File 14). POPA1 contained 12 citrus SNPs; these were SNPs 1_1415 to 1_1426 (further details not included).

**POPA2 SNP Selection**

The same three sources of SNPs for POPA1 were used also for POPA2, but the selection and prioritization methods for POPA2 differed from POPA1. SNPs with Illumina SNP design score of 0.4 or higher were accepted, rather than 0.59 applied to POPA1; this increased the number of SNPs under consideration. The UCR EST-SNP algorithm was adjusted to exclude EST sequence positions within 40 bases of polyA ends or polyT beginnings; this reduced the number of UCR SNPs under consideration. The content of POPA2 included all remaining, but was not confined to, stress-related SNPs in the UCR SNP list; this increased the number of SNPs under consideration. Also, 258 POPA2 SNPs were from genes previously targeted on POPA1; this reduced the number of newly represented genes to 1278 (1536 minus 258). Positive prioritization factors that influenced the final content of POPA2 were: 1) the SNP tested on POPA1 had a low GenTrain score (208 POPA2 SNPs), chosen to have a second try for high technical success; 2) the SNP tested on POPA1 had a high GenTrain score (50 POPA 2 SNPs), chosen to enable more haplotype sensitivity for certain genes; 3) the position in the genome was of special interest or not well saturated among POPA1 SNPs based on barley/rice synteny, 4) the SNP came from a SCRI or IPK amplicon sequence alignment, or 5) the barley gene had no apparent rice homolog. SNPs that were supported only by Morex-Morex or Barke-Barke comparisons were downweighted in POPA2 SNP selection. The same SCRI, IPK and UCR name conventions apply to the original SNP names on POPA2 as POPA1 (see above). The final composition of POPA2 included 1536 barley SNPs originating from 1456 UCR, 59 SCRI and 21 IPK SNPs. The original SNP names and a simplified naming convention, 2_0001 through 2_1536, are included in Table S4 (Additional File 14).

**BOPA1 SNP Selection**

BOPA1 represents 705 SNPs from POPA1 and 832 from POPA2, including one SNP in common. All BOPA1 SNPs had a satisfactory technical performance on POPA1 or POPA2 and a homozygous major allele frequency of not more than 0.92 within germplasm samples that were applied to POPA1 and POPA2 (NR, TJC, SC). Since heterozygotes were very rare, this means that with only a few exceptions BOPA1 SNPs had a minor allele frequency of at least 0.08. At the time when BOPA1 was designed, BOPA1 included 1314 mapped and 222 unmapped SNPs. To the extent of results presented in this paper, BOPA1 included 1414 mapped and 122 unmapped SNPs; the additional 100 mapped SNPs are attributed mainly to the Haruna Nijo x OHU602 mapping population (see below). Two name conventions for BOPA1 SNPs are included in Table S4 (Additional File 14), either 11_0001 through 11_1536, which derive from alphanumeric sorting of the original SNP names, or 11_ followed by a five digit concatenation of the POPA name. For example POPA2 SNP 2_0606 has a BOPA1 concatenated name of 11_20606. BOPA1 represents 1312 UCR, 169 SCRI and 55 IPK SNPs.

**POPA3 SNP Sources and Selection**

Residual SNPs from the three sources for POPA1 and POPA2 were used for POPA3, but additional SNPs were required. Additional SNPs came from three sources: 1) an extended list of 5732 SNPs from SCRI (NR) derived from reanalysis of the amplicon sequence alignments used for POPA1 and POPA2, 2) a new HarvEST:Barley CAP3 relaxed assembly (#35) containing 444,652 sequences from 323,165 clones (TJC, SW), and 3) colleagues who contributed SNPs from sequence alignments of alleles of specific genes of biological interest. HarvEST:Barley assembly #35 included additional EST sequences provided by authors KS and NS. This yielded 14,601 SNPs in 4415 assembly #35 unigenes from 253 pairwise comparisons between ESTs from 23 genotypes using the SNP finding method described above, except that version 4 of the rice genome was utilized to mark intron positions. Individuals who provided additional SNPs were authors LR and AD (242 SNPs in 94 genes; SCRI), authors PS and PH (372 SNPs in 60 genes; Oregon State University); Peter Morrell (500 SNPs in 17 genes; UC Irvine); Hatice Bilgic and Brian Steffenson (31 SNPs in the *Rpg1* gene; U Minnesota), and Roger Wise and author MJM (331 SNPs in the *Mla* gene; Iowa State University). The names of SCRI SNPs from reanalyzed amplicon alignments begin with ABC and the IPK names are as in POPA1 and POPA2 (ConsensusGBS). UCR SNPs from assemblies #32 and #35 are distinguished by the prefix U32_ or U35_ followed by unigene_position. All other SNPs begin with the institutional letters of contributing colleagues (ISU, OSU, SCRI, UM, UCI). These original SNP names and a simplified naming convention, 3_0001 through 3_1536, are included in Table S4 (Additional File 14). The relationships of all but a few POPA3 SNPs to assembly #35 (and #32) unigenes are included in Table S4 (Additional File 14). Selection of SNPs for POPA3 proceeded as follows. SNPs previously represented on POPA1 or POPA2 were excluded. SNPs with an Illumina SNP score less than 0.4 were excluded. A total of 810 SNPs from the three sources for POPA1 and POPA2 plus the extended SNP list from SCRI were included; this was 301 SCRI, 1 IPK and 508 UCR SNPs. A total of 267 SNPs targeting specific genes in the lists from colleagues were included, generally more than one SNP per gene. This was 7 ISU, 86 OSU, 120 SCRI, 48 UCI and 6 UM SNPs. Finally, 459 UCR SNPs from assembly #35 were included to top up to 1536, targeting genes not otherwise represented on POPA1, POPA2 or the remainder of POPA3. In this final set, priority was given to genes previously classified by Nora Lapitan (Colorado State University) and Blake Cooper (Annheuser-Busch) as having interesting expression patterns during malting, or by Roger Wise (Iowa State University) or author GJM as having interesting expression patterns upon exposure to pathogens, or by author PH (Oregon State University) as relevant to malting, brewing quality, abiotic stress or phenology.

**BOPA2 SNP Selection**

BOPA2 represents 406 SNPs from POPA1, 178 from POPA2 and 952 from POPA3. As with BOPA1, only SNPs with satisfactory technical performance were selected for BOPA2. However, unlike BOPA1 which was restricted to SNPs with high MAF, the primary emphases of BOPA2 were representation of mapped SNPs that were not included on BOPA1 and inclusion of multiple SNPs for certain genes to reveal haplotypes at these loci, with some consideration of MAF. To the extent of results presented in this paper, BOPA2 included 1263 mapped and 273 unmapped SNPs. BOPA2 contained 921 SNPs with minor allele frequency (MAF) at least 0.08, 256 SNPs with MAF at least 0.04 but less than 0.08, 345 SNPs with MAF least 0.005 but less than 0.04, and 14 SNPs that had only one allele (MAF = 0) in the germplasm examined using POPA3 but were included in BOPA2 because of additional knowledge. Like BOPA1, alternative names for BOPA2 SNPs are included in Table S4 (Additional File 14), either 12_ followed by a concatenation of the POPA name, or 12_0001 through 12_1536 from alphanumeric sorting of the concatenated POPA SNP names. BOPA2 was composed of SNPs from the following sources (mean MAF indicated in parentheses): 967 from UCR assemblies #32 and #35 (MAF = 0.186), 412 from SCRI (MAF = 0.148), 76 from OSU (MAF = 0.221), 52 from IPK (MAF = 0.058), 23 from UCI (MAF = 0.138), 5 from UM (MAF = 0.140), 1 from ISU (MAF = 0.280).

**SNP annotations**

Table S4 (Additional File 14) provides alternative SNP names arising from this work, and annotation fields for all SNPs represented on POPA1, POPA2, POPA3, BOPA1 and BOPA2. Annotations include assembly #32 and #35 unigene, Affymetrix Barley1 GeneChip probe set(s) matching the unigene, the best BLAST hits to rice, Arabidopsis and UniProt, the position of each mapped gene by chromosome, map location, and the consensus sequence of the unigene which served as the source sequence. The results of work to be described elsewhere (Bhat et al., in preparation) following the method described in Simkova et al. [24], delimited the centromere position and provided chromosome arm assignments used for Fig. 6. The annotation information in Table S4 (Additional File 14) for 2943 mapped SNPs (see below) is available from HarvEST:Barley [14, 20]. The HarvEST BLAST server [25] provides the mapped SNP unigenes as a searchable database.

**DNA sources**

Genomic DNAs of 93 doubled haploid maplines and the parents (Dom, Rec) of the Oregon Wolfe Barley (OWB) population [26] [27], 148 doubled haploids and the parents of the Steptoe x Morex (SxM) population [7] [28], and 213 additional germplasm samples were purified using Plant DNeasy (Qiagen, Valencia, CA, USA) starting with 100-300 mg of young seedling leaves. Seeds of OWB and SxM maplines were provided in the mid-1990s by author PH (Oregon State University) and periodically re-grown at UC Riverside (RDF, TJC), where their DNAs were produced for the work described here. Germplasm DNA samples were collected at UC Riverside after production using the same Plant DNeasy method as stated above at three locations: 1) SCRI from SCRI and IPK seed stocks (NR), 2) Oregon State University (PH) and 3) UC Riverside (RDF, JS, TJC, PB). Genomic DNAs of 93 doubled haploid maplines and the Barke parent from the Morex x Barke population were produced at IPK Gatersleben using a CTAB method and sent to UC Riverside (NS). All of the above DNA samples were checked for DNA concentration using UV spectroscopy and Quant-iT PicoGreen (Invitrogen, Carlsbad, CA, USA) and adjusted to approximately 120 ng/µl in TE buffer at UC Riverside (RDF with assistance of Jayati Mandal) before transporting to the genotyping facility at UC Los Angeles (UCLA). DNAs from doubled haploid maplines and the parents of the Haruna Nijo x OHU602 (HxO) population were prepared at Okayama University (KS) and sent directly to the UCLA genotyping facility.

**Data production for map construction and MAF estimation**

DNA Concentrations were re-checked using Quant-iT PicoGreen (Invitrogen, Carlsbad, CA) and standardized to 80 ng/µl in TE buffer in preparation for the GoldenGate assay (author JD with assistance of Maricel Almonte and Oi-wa Choi). 5 µl (400 ng) were used for each assay. Data were generated from each progeny line in the OWB, SxM and MxB doubled haploid populations using POPA1 and POPA2. Data were also produced using POPA3 from the complete OWB and MxB sets of DNA samples, but from only 92 SxM doubled haploids. Data from 95 HxO doubled haploids using BOPA1 were also included. For each of these four mapping populations, extensive integration of SNP data with other types of marker data will be described elsewhere (for example OWB marker integration in Szűcs et al. [10]). Data used for the determination of allele frequency (see below) came from 125 germplasm samples for POPA1, 195 germplasm samples for POPA2, and 189 germplasm samples for POPA3.

**Data processing**

Raw data were transformed to genotype calls, initially using Illumina GenCall and subsequently using Illumina BeadStudio version 3 with the genotyping module. For each OPA, the data from all samples were visually inspected in order to manually set 1536 archetypal clustering patterns. The cluster positioning was guided by knowledge that heterozygotes are nearly non-existent in doubled haploids and rare in highly inbred parental genotypes and germplasm samples. Several “synthetic heterozygote” DNA samples were made by mixing parental DNAs in a 1:1 mass ratio (Fig. 2A, green dots), and included to anchor heterozygote cluster positions which enable the identification of true heterozygotes which occur at a significant frequency in germplasm samples that have not been sufficiently inbred to reach a state of genome-wide allele fixation. Polymorphisms with theta compressed clusters were not used if the compression was such that any alternative homozygote calls were not clearly distinguishable from each other (Fig. 2B, set as Gentrain 0.000, 100% “no call”). Also, vertically separated data clusters, even when clearly separated from each other, were not accepted as polymorphisms (Fig. 2C, set as Gentrain 0.000, 100% “no call”). The spatial positions of heterozygote and homozygote data clusters were in all cases confined to areas of high certainty so that data points with less certainty outsides the boundaries of heterozygotes and homozygotes were scored as “no-call” (Fig 2A, one germplasm sample as black dot). Genotype calls were exported as spreadsheets from BeadStudio. The no-call threshold was set to 0.15; this necessitated a manual override of the genotype call exported from the Bead Studio software in cases which were plainly evident by eye and not in conflict with the genetic map. Following the production of one master workspace for each Pilot OPA using all DNA samples, customized workspaces were produced for each mapping population to further optimize the genotype calls using minor adjustments of the cluster positions.

**Individual and consensus map production**

Individual maps were made principally using MSTMap [29] [30] for each data set from the four doubled haploid mapping populations (PB, YW, SB, SL). In brief, MSTMap first identifies linkage groups, then determines marker order by finding the minimum spanning tree of a graph for each linkage group, then calculates distances between marker using recombination frequencies. JoinMap 4 [31] was used to confirm linkage groups and marker order determined by MSTMap (PB, MLR). MapInspect [39 ] and Microsoft Access and Excel were used to visualize relationship between maps made using different algorithms (PB, MLR, TJC). Raw data for problematic markers were reviewed using BeadStudio and then their genotype calls were either discarded entirely or readjusted when it was plainly evident that such adjustments were warranted. Each such review was followed by the production of new maps; this iterative process generally involved 10-20 cycles for each individual map. At several points in the mapping, a consensus map was produced using MergeMap [32] which also flags problematic markers (YW, SL, PB). MergeMap takes into account marker order from individual maps and calculates a consensus marker order. Briefly, the input to MergeMap is a set of directed acyclic graphs (DAGs) from each linkage group of each individual map [33], where each DAG is consistent with all (or nearly all) of the markers in the individual input maps. MergeMap computes the consensus DAGs (Fig. 4, Figures S3-S9, Additional Files 6 - 12) by formulating the optimization problem of resolving ordering conflicts as an integer linear program. MergeMap then linearizes each consensus DAG using a mean distance approximation. The consensus map coordinates from MergeMap were normalized to the arithmetic mean cM distance for each linkage group from the four individual maps (TJC).

**Implementation of BOPA1 and BOPA2 in US barley breeding germplasm**

As part of Barley CAP [17], the two BOPAs are being used to genotype a total of 3840 US barley breeding lines contributed from ten US barley breeding programs for association mapping analyses. For this work, the GoldenGate assay is carried out in the USDA-ARS small grains genotyping center directed by author SC in Fargo, ND. As of January 2009, data from both BOPAs had been generated for 1920 breeding lines, with 960 submitted for each of two years, 2006 and 2007. Before releasing genotyping data to the breeders, raw data files were jointly evaluated in the Fargo and Riverside locations using BeadStudio 3. To maximize the consistency of the data processing path, raw data were pooled for all 1920 samples for each BOPA. Prior knowledge of data clustering patterns and the technical behavior of each SNP on the pilot OPAs were consulted to assist in resolving uncertainties encountered in the use of the BOPAs (SC, PB, TJC).

**Alternative marker names**

Cross-referencing to the GrainGenes “Sequenced Probes” database was done by DEM. Further cross-referencing for Table S3 (Additional File 13) was by TJC. The bin numbers for 110 markers from Kleinhofs and Graner [11] were provided for Table S1 (Additional File 3) by AK.
