# Supplementary figures and images for: Development and implementation of high-throughput SNP genotyping in barley
Source: BMC Genomics. 2009 Dec 4;10:582. doi: 10.1186/1471-2164-10-582 (PMC2797026; doi:10.1186/1471-2164-10-582)

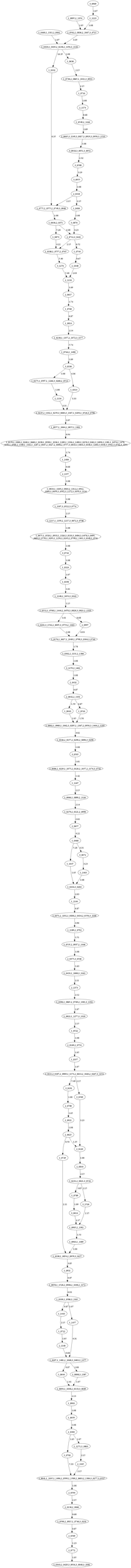

Supplement: Additional file 6 — Figure S3. Complete consensus directed acyclic graphs for barley chromosomes 1H. [file 1471-2164-10-582-S6.JPEG]

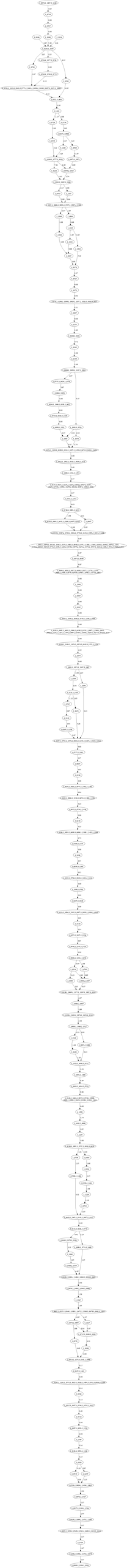

Supplement: Additional file 7 — Figure S4. Complete consensus directed acyclic graphs for barley chromosomes 2H. [file 1471-2164-10-582-S7.JPEG]

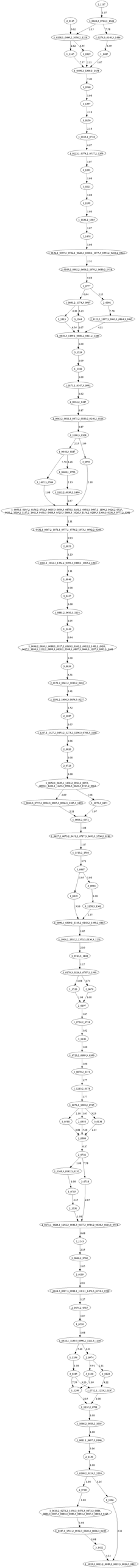

Supplement: Additional file 9 — Figure S6. Complete consensus directed acyclic graphs for barley chromosomes 4H. [file 1471-2164-10-582-S9.JPEG]

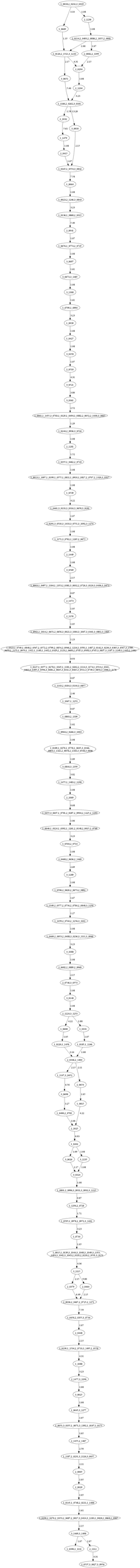

Supplement: Additional file 11 — Figure S8. Complete consensus directed acyclic graphs for barley chromosomes 6H. [file 1471-2164-10-582-S11.JPEG]

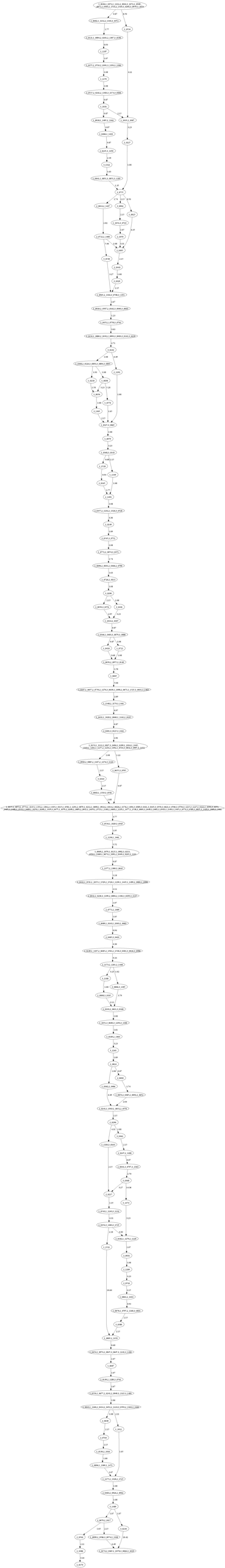

Supplement: Additional file 12 — Figure S9. Complete consensus directed acyclic graphs for barley chromosomes 7H. [file 1471-2164-10-582-S12.JPEG]
