# Supplementary material for: Development and implementation of high-throughput SNP genotyping in barley
Source: BMC Genomics. 2009 Dec 4;10:582. doi: 10.1186/1471-2164-10-582 (PMC2797026; doi:10.1186/1471-2164-10-582)
Supplement: Additional file 13 — Table S3. Synonymous marker names. [file 1471-2164-10-582-S13.DOC]

Supplemental Table S3. Synonymous marker names.

| **U35** | **Marcel Probe** | **Marcel Locus** | **Chrom** | **cM Marcel** | **Stein** | **cM This Work** | **Arm** | **POPA** | **BOPA** | **BOPA_C** | **Original SNP Name** |
| --- | --- | --- | --- | --- | --- | --- | --- | --- | --- | --- | --- |
| 904 | ABG319 | ABG319e | 1H | 16.65 |  | 10.67 | S | 3_1533 |  |  | U35_904_404 |
| 16128 | MWG913 | MWG913 | 1H | 57.78 |  | 42.52 | S | 1_0597 | 11_0648 | 11_10597 | 409-1643 |
| 8281 | ABG494 | ABG494 | 1H | 61.06 |  | 49.70 | S | 3_0258 |  |  | ABC16875_1_129 |
| 17958 | cMWG758 | cMWG758 | 1H | 60.52 | 1 | 55.49 | L | 3_0110 | 12_0642 | 12_30110 | ABC08981_1_44 |
| 5111 | MWG800 | MWG800 | 1H | 65.87 |  | 59.71 | L | 2_1333 | 11_1218 | 11_21333 | 8486-1964 |
| 1948 | ABG373 | ABG373 | 1H | 136.75 |  | 135.56 | L | 2_0603 | 11_0564 | 11_20603 | 3671-59 |
| 1739 | ABG387 | ABG387a | 1H | 138.75 |  | 138.92 | L | 2_0509 | 11_0485 | 11_20509 | 3246-1135 |
| 1246 | cMWG682 | cMWG682 | 2H | 8.11 | 1 | 6.45 | S | 1_0326 | 11_0363 | 11_10326 | 2582-767 |
| 15221 | cMWG655 | cMWG655 | 2H | 24.09 | 1 | 10.06 | S | 2_0261 |  |  | 1870-1887 |
| 15481 | MWG874 | MWG874 | 2H | 54.91 | 1 | 38.48 | S | 1_1073 | 11_1333 | 11_11073 | ABC03253-1-2-279 |
| 21741 | CDO537 | CDO537 | 2H | 66.48 |  | 56.28 | S | 3_0251 | 12_0732 | 12_30251 | ABC15603_1_183 |
| 1284 | cMWG699 | cMWG699 | 2H | 84.06 | 1 | 85.92 | L | 1_0287 | 11_0320 | 11_10287 | 2371-950 |
| 605 | CDO373 | CDO373 | 2H | 118.53 |  | 127.64 | L | 1_0109 | 11_0154 | 11_10109 | 1486-908 |
| 605 | CDO373 | CDO373 | 2H | 118.53 |  | 127.64 | L | 2_0183 | 12_0426 | 12_20183 | 1486-1515 |
| 6860 | CDO36 | CDO036 | 2H | 144.83 |  | 152.79 | L | 3_1506 | 12_1518 | 12_31506 | U35_6860_433 |
| 21052 | MWG2158 | MWG2158 | 3H | 13.76 |  | 6.03 | S | 1_1453 | 11_1494 | 11_11453 | ConsensusGBS0194-1 |
| 15527 | ABG471 | ABG471 | 3H | 33.14 |  | 35.22 | S | 3_0431 | 12_0828 | 12_30431 | U32_2436_2433 |
| 16766 | ABG398 | ABG398 | 3H | 48.42 |  | 61.44 | L | 1_0005 | 11_0010 | 11_10005 | 10126-999 |
| 15500 | cMWG680 | cMWG680 | 3H | 58.01 | 1 | 64.19 | L | 1_0281 | 11_0315 | 11_10281 | 2338-1572 |
| 259 | BCD263 | BCD263 | 3H | 60.6 |  | 65.52 | L | 3_0005 | 12_0589 | 12_30005 | ABC01483_1_288 |
| 2806 | PSR156 | PSR156 | 3H | 64.92 |  | 73.53 | L | 3_1356 | 12_1431 | 12_31356 | U35_2806_883 |
| 14619 | CDO1406 | CDO1406 | 3H | 70.33 |  | 78.53 | L | 1_0047 | 11_0067 | 11_10047 | 1176-1547 |
| 5138 | CDO669 | CDO669A | 4H | 19.75 |  | 20.12 | S | 2_1359 | 11_1241 | 11_21359 | 8724-1282 |
| 2593 | ABG397 | ABG397 | 4H | 103.64 |  | 42.32 | S | 2_0770 | 12_0494 | 12_20770 | 4586-536 |
| 1724 | CDO348 | CDO348B | 5H | 57.08 |  | 69.90 | L | 1_0641 | 11_0707 | 11_10641 | 4342-528 |
| 14265 | PSR128 | PSR128 | 5H | 66.62 |  | 80.61 | L | 1_0127 | 11_0175 | 11_10127 | 1583-522 |
| 14926 | ABC302 | ABC302 | 5H | 66.62 |  | 80.61 | L | 2_0236 | 11_0200 | 11_20236 | 171-1301 |
| 15957 | WG364 | WG364 | 5H | 90.08 |  | 103.92 | L | 1_0414 | 11_0449 | 11_10414 | 3056-1317 |
| 14983 | cMWG781 | cMWG781 | 5H | 109.51 | 1 | 127.24 | L | 2_0405 |  |  | 2737-1073 |
| 17977 | CDO504 | CDO504 | 5H | 121.33 |  | 134.60 | L | 2_0487 | 11_0462 | 11_20487 | 314-559 |
| 15551 | cMWG654 | cMWG654 | 5H | 144.93 | 1 | 159.79 | L | 1_0385 | 11_0412 | 11_10385 | 2867-373 |
| 16073 | CDO484 | CDO484 | 5H | 170.96 |  | 182.16 | L | 3_0504 | 12_0865 | 12_30504 | U32_3706_309 |
| 811 | PSR167 | PSR167A | 6H | 3.81 |  | 0.00 | S | 2_0232 | 11_0197 | 11_20232 | 1692-742 |
| 2587 | MWG2202 | MWG2202 | 6H | 5.36 |  | 1.34 | S | 2_0886 | 11_0844 | 11_20886 | 5159-579 |
| 18664 | ABG466 | ABG466 | 6H | 9.24 |  | 3.23 | S | 2_1199 |  |  | 7135-1137 |
| 18027 | MWG573 | MWG573 | 6H | 15.39 |  | 9.06 | S | 2_1032 | 11_0967 | 11_21032 | 5993-2383 |
| 15930 | ABG654 | ABG654B | 6H | 18.46 |  | 16.97 | S | 1_0554 | 12_0128 | 12_10554 | 384-815 |
| 15930 | ABG654 | ABG654B | 6H | 18.46 |  | 16.97 | S | 3_0842 | 12_1080 | 12_30842 | OSU_HA16L09r_s_at_77 |
| 4048 | CDO497 | CDO497 | 6H | 61.36 |  | 55.65 | L | 2_1216 | 11_1116 | 11_21216 | 7282-1386 |
| 15282 | ABC163 | ABC163 | 6H | 71.66 |  | 64.36 | L | 2_0287 | 11_0249 | 11_20287 | 2026-302 |
| 1958 | ABC175 | ABC175 | 6H | 72.02 |  | 70.04 | L | 2_0620 | 11_0589 | 11_20620 | 3773-756 |
| 15893 | ABC154 | ABC154C | 6H | 99.96 |  | 104.27 | L | 2_0464 |  |  | 3029-1441 |
| 15384 | BCD276 | BCD276 | 6H | 115.01 |  | 112.32 | L | 1_0239 | 11_0275 | 11_10239 | 2152-1547 |
| 15758 | cMWG684 | cMWG684a | 6H | 139.9 | 1 | 128.48 | L | 1_0390 | 11_0421 | 11_10390 | 2911-1136 |
| 14496 | cMWG703 | cMWG703 | 7H | 24.66 | 1 | 21.13 | S | 1_0025 | 11_0036 | 11_10025 | 1073-916 |
| 18161 | ABC158 | ABC158 | 7H | 41.28 |  | 38.32 | S | 1_0838 | 11_1010 | 11_10838 | 6353-524 |
| 14484 | ABC255 | ABC255 | 7H | 60.99 |  | 60.69 | S | 1_1014 | 11_1280 | 11_11014 | 943-3107 |
| 14484 | ABC255 | ABC255 | 7H | 60.99 |  | 61.32 | S | 3_0879 | 12_1114 | 12_30879 | OSU_SS1_129 |
| 14484 | ABC255 | ABC255 | 7H | 60.99 |  | 61.32 | S | 3_0880 | 12_1115 | 12_30880 | OSU_SS1_201 |
| 14731 | HVCMA | HVCMA | 7H | 67.59 |  | 62.88 | S | 1_0721 | 11_0808 | 11_10721 | 497-386 |
| 14851 | cMWG725 | cMWG725 | 7H | 82.26 | 1 | 74.52 | S | 2_0885 | 11_0843 | 11_20885 | 5-1593 |
| 15785 | MWG626 | MWG626 | 7H | 79.79 |  | 77.85 | S | 1_0394 | 11_0423 | 11_10394 | 2924-1189 |
| 15408 | MWG957 | MWG957 | 7H | 82.26 |  | 79.60 | L | 1_0713 | 12_0171 | 12_10713 | 486-1812 |
| 15408 | MWG957 | MWG957 | 7H | 82.26 |  | 79.60 | L | 3_0835 | 12_1073 | 12_30835 | OSU_Contig742_at_365 |
| 755 | PSR129 | PSR129 | 7H | 110.8 |  | 121.09 | L | 2_0217 | 12_0433 | 12_20217 | 1621-2223 |

**HarvEST:Barley assembly #35 unigene, probe and locus in Marcel et al. [3], chromosome (all maps agree), map position in Marcel et al. [3], “1” if the same marker is in Stein et al. [4], map position in the present work, arm as described in the text, name for Pilot OPA, initial and concatenated name for production OPA, original name (see Supplemental Text for more details on SNP names).**
