# Supplementary material for: Development and implementation of high-throughput SNP genotyping in barley
Source: BMC Genomics. 2009 Dec 4;10:582. doi: 10.1186/1471-2164-10-582 (PMC2797026; doi:10.1186/1471-2164-10-582)
Supplement: Supplementary file 6 [file 1471-2164-10-582-S2.DOC]

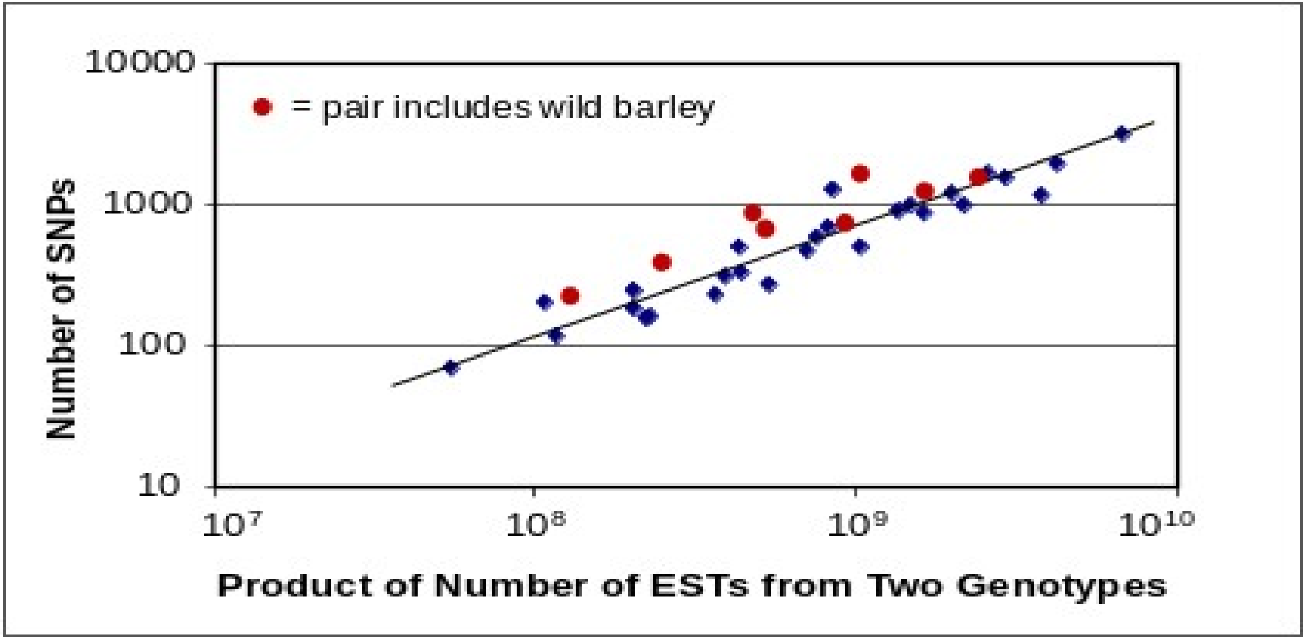

10000
1000
100
10
= pair includes wild barley
Number of SNPs
10^7
10^8
10^9
10^10
Product of Number of ESTs from Two Genotypes
