# Supplementary material for: Development and implementation of high-throughput SNP genotyping in barley
Source: BMC Genomics. 2009 Dec 4;10:582. doi: 10.1186/1471-2164-10-582 (PMC2797026; doi:10.1186/1471-2164-10-582)
Supplement: Supplementary file 7 [file 1471-2164-10-582-S4.DOC]

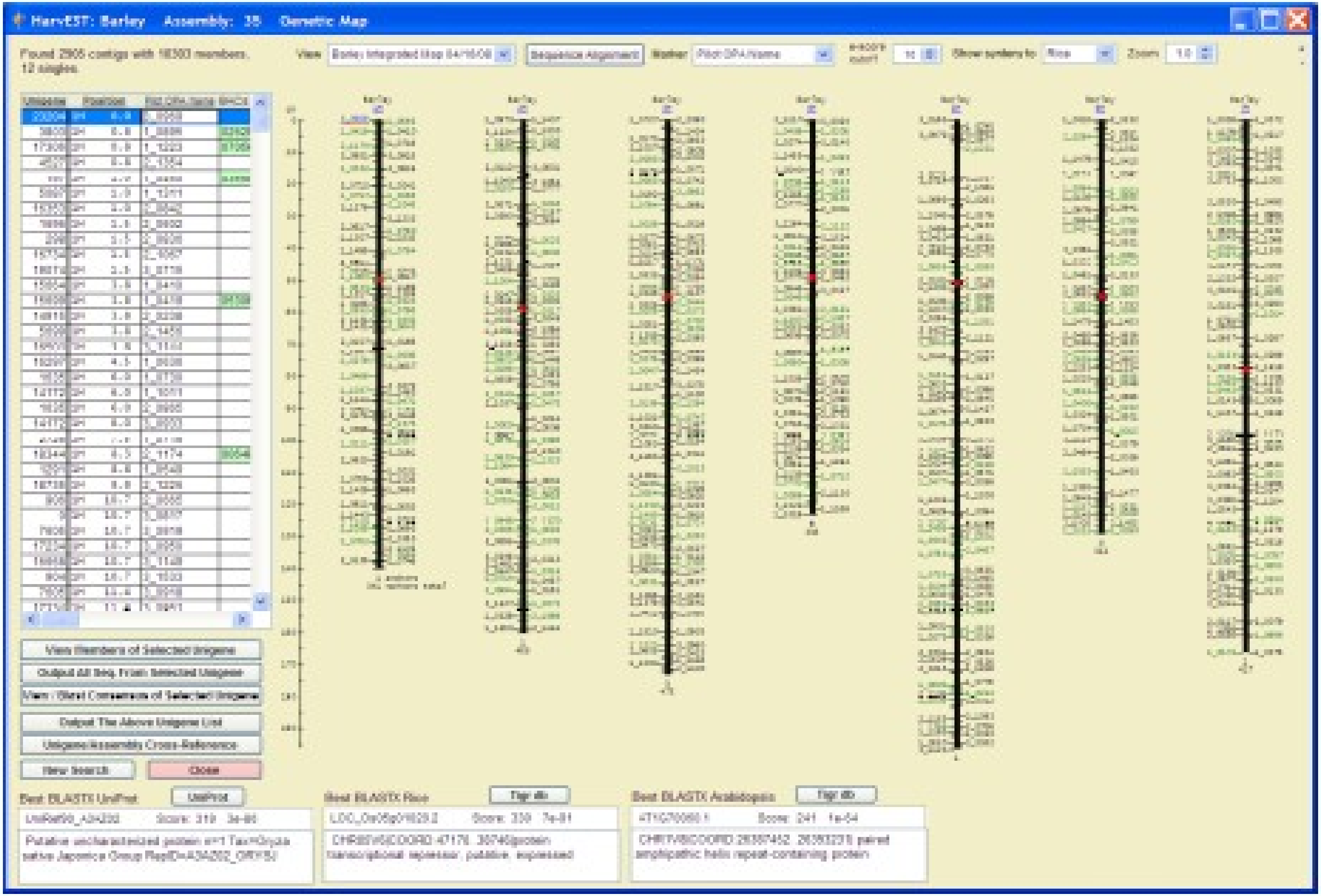
HarvEST: Barley Assembly: 35 Genetic Map
View
Sequence Alignment
Marker
Show synteny to
Rice
Zoom
View Members of Selected Unigene
Output All Seq. From Selected Unigene
View / Blast Consensus of Selected Unigene
Output The Above Unigene List
Unigene/Assembly Cross-Reference
New Search
Close
Best BLASTX UniProt
UniProt
Best BLASTX Rice
Top db
Best BLASTX Arabidopsis
Top db
